# Color Image Encryption Based on 3D-SBFCM with Dynamic Rectangular Partitioning and Dynamic S-Box Substitution

**DOI:** 10.3390/e28060653

**Published:** 2026-06-09

**Authors:** Ting Wang, Xiaoyan Yang, Bin Ge, Chenxing Xia, Houyue Wu

**Affiliations:** 1 School of Information Engineering, Huainan Union University, Huainan 232038, China; yangxiaoyan@hnuu.edu.cn; 2 School of Computer Science and Engineering, Anhui University of Science and Technology, Huainan 232001, China; bge@aust.edu.cn (B.G.); cxxia@aust.edu.cn (C.X.); 3 State Key Laboratory of Digital Intelligent Technology for Unmanned Coal Mining, Anhui University of Science and Technology, Huainan 232001, China; 4 School of Computer and Information Engineering, Bengbu University, No. 1866 Caoshan Road, Bengbu 233030, China; why@bbc.edu.cn

**Keywords:** color image encryption, chaotic map, hierarchical scrambling, dynamic S-box, cross-channel diffusion

## Abstract

Existing chaos-based color image encryption algorithms still face several challenges, including insufficient dynamical complexity of low-dimensional chaotic maps, residual boundary regularity caused by fixed block partitioning, and limited diffusion among RGB channels. To address these issues, this paper proposes a color image encryption algorithm based on a three-dimensional sine-bilinear fully coupled chaotic map (3D-SBFCM). The proposed map integrates sinusoidal modulation, linear coupling, and bilinear cross-coupling within a mod-1 mapping framework, thereby improving the complexity and pseudorandomness of the generated chaotic sequences. In addition, a residual-feasibility-constrained dynamic rectangular partitioning mechanism is developed to generate reversible non-uniform image blocks and reduce the structural regularity associated with fixed-size partitioning. Based on this partitioning structure, inter-block permutation among same-size blocks and intra-block two-dimensional permutation are performed to weaken both global and local spatial correlations. Plaintext-related initialization, dynamic S-box substitution, and forward-backward cross-channel diffusion are further incorporated into the overall permutation-diffusion framework to enhance plaintext sensitivity, nonlinear confusion, and perturbation propagation across RGB channels. Experimental results demonstrate that the proposed algorithm effectively conceals the statistical characteristics of plaintext images, with information entropy values higher than 7.999 for all color channels and NPCR/UACI values close to their theoretical expectations. The algorithm also shows satisfactory robustness against cropping and noise attacks. These results indicate that the proposed method provides an effective and secure solution for color image encryption.

## 1. Introduction

Images are characterized by high redundancy, strong spatial correlation, and large data volume [1,2], which makes image encryption fundamentally different from conventional text encryption. A secure image cipher should conceal visual information, suppress statistical features, and reduce correlations among neighboring pixels. Owing to their sensitivity to initial conditions, ergodicity, aperiodicity, and pseudo-random behavior, chaotic systems have been widely used in image encryption [3,4,5,6]. Early studies mainly adopted low-dimensional maps, such as the logistic map [7,8], sine map [9], and Henon map [10,11]. Although these maps are easy to implement, their simple structures usually lead to narrow chaotic intervals, limited key spaces, uneven sequence distributions, and weak resistance to statistical analysis, especially in color image encryption requiring strong plaintext sensitivity and inter-channel diffusion.

To improve classical low-dimensional maps, researchers have introduced nonlinear reconstruction, parameter extension, hybrid coupling, and bit-level perturbation. Sun et al. [12] combined Logistic and Sine maps with cellular automata for color image encryption. Dua et al. [13] introduced fractional-order operations into a cosine-based map to enlarge the key space. Li et al. [14] proposed an enhanced logistic modular map using modular and exponential nonlinearities. Zhao et al. [15] constructed a Henon-Sine coupled map, whereas Sharma et al. [16] employed XOR-based bit rearrangement to extend the chaotic behavior of the Henon map. These methods improve sequence complexity to some extent; however, many of them still suffer from limited state interaction, making it difficult to provide sufficient global disturbance for complex color images.

High-dimensional and hyperchaotic systems have also attracted considerable attention. Zhang et al. [17] developed a four-dimensional hyperchaotic system based on the Chen system and combined it with SHA-384 to increase plaintext dependence. Alexan et al. [18] used a five-dimensional chaotic system and nonlinear transformations to expand the key domain. Sun et al. [19] proposed a six-dimensional non-degenerate discrete hyperchaotic system with compressed sensing and plaintext-related scrambling. Kaur et al. [20] applied a seven-dimensional chaotic system to lightweight biomedical image protection. Compared with low-dimensional maps, these systems generally exhibit richer dynamics and stronger parameter sensitivity. Nevertheless, high-dimensional continuous systems often involve multiple parameters and numerical integration, resulting in higher computational cost and implementation complexity. This limits their deployment in real-time or resource-constrained scenarios, such as mobile devices and IoT edge terminals [21].

Meanwhile, chaos-based encryption has been combined with DNA coding, blockchain, artificial intelligence, and other techniques. Chen et al. [22] integrated an improved Arnold map, dynamic DNA encoding, and a dual hyperchaotic system to reduce pixel correlation. Kumaran et al. [23] combined DNA operations with elliptic curve cryptography for medical image encryption. Neela et al. [24] proposed a blockchain-driven private-key generation scheme, while Inam et al. [25] introduced blockchain-assisted signature verification into chaotic medical image transmission. Ahn and Hong [26] designed a quantum GAN-based key generator, and Nair et al. [27] combined hyperchaotic maps, memristive maps, and convolutional neural networks. These methods can enhance security to some extent, but they may also increase computational complexity, storage overhead, communication cost, or implementation difficulty. The review studies by Li et al. [28] and Gao et al. [29] also pointed out that chaos-based encryption algorithms should not only focus on statistical randomness, but also comprehensively consider the balance among security, complexity, implementation efficiency, and practical application scenarios.

In addition to chaotic sequences themselves, permutation and diffusion structures are also important factors affecting the security of image encryption. Many image encryption algorithms adopt a permutation-diffusion structure, where permutation is used to change pixel positions and diffusion is used to change pixel values. Fixed-size block scrambling has the advantages of simple implementation and high computational efficiency. However, its block boundaries are usually located at fixed row and column intervals. For example, 8 × 8 or 16 × 16 block partitioning forms a regular grid structure. Even after inter-block permutation, such fixed grid boundaries may still retain certain structural regularity, which is referred to in this paper as residual boundary regularity caused by fixed block partitioning. Therefore, relying only on fixed-size block partitioning may be insufficient to fully destroy the spatial structure of an image.

Moreover, some color image encryption algorithms process the R, G, and B channels separately, or introduce only weak inter-channel diffusion. When a slight pixel change occurs in one channel, the perturbation may not sufficiently propagate to the other channels or the entire cipher image, thereby affecting the algorithm’s resistance to differential attacks. Therefore, color image encryption algorithms need to jointly consider non-uniform spatial scrambling, nonlinear substitution, and cross-channel diffusion.

In summary, existing methods still have the following limitations. First, some low-dimensional chaotic maps have simple structures and high efficiency, but their dynamical complexity and state coupling capability are insufficient. Second, high-dimensional or continuous hyperchaotic systems exhibit richer dynamical behaviors, but they usually involve higher computational complexity and implementation cost. Third, hybrid schemes based on DNA coding, blockchain, artificial intelligence, and other mechanisms can enhance security, but they may introduce additional overhead. Fourth, fixed-size block partitioning tends to produce regular boundaries and may retain grid-like structural regularity. Fifth, the diffusion capability among RGB channels still needs to be further strengthened.

Based on these considerations, this paper adopts a three-dimensional sine-bilinear fully coupled chaotic map to generate pseudorandom sequences and constructs the encryption algorithm from three aspects: permutation, substitution, and diffusion. Specifically, dynamic rectangular block partitioning is used to reduce the residual boundary regularity caused by fixed block partitioning; a dynamic S-box is introduced to enhance nonlinear confusion; and a forward-backward cross-channel diffusion mechanism is designed to strengthen disturbance propagation among RGB channels. The main contributions are summarized as follows:

(1) A new 3D-SBFCM is constructed by integrating sinusoidal modulation, linear coupling, and bilinear cross-coupling into a discrete mod-1 map, thereby improving the complexity and pseudorandomness of the generated chaotic sequences.

(2) A residual-feasibility-constrained dynamic rectangular partitioning strategy is proposed to generate reversible non-uniform image blocks and reduce the boundary regularity caused by fixed-size partitioning.

(3) Based on the proposed partitioning structure, same-size inter-block permutation and intra-block two-dimensional permutation are performed to weaken global and local spatial correlations while maintaining block-boundary compatibility.

(4) Plaintext-related initialization, dynamic S-box substitution, and forward–backward cross-channel diffusion are incorporated into the permutation-diffusion framework to enhance plaintext sensitivity, nonlinear confusion, and RGB-channel perturbation propagation.

## 2. Construction and Analysis of the Proposed 3D-SBFCM

This section presents the construction of the proposed three-dimensional sine-bilinear fully coupled chaotic map (3D-SBFCM) and then analyzes its dynamical behavior and pseudorandomness.

### 2.1. Classical One-Dimensional Mod-1 Map

The classical one-dimensional mod-1 map is a nonlinear discrete system defined by the modulo operation. It is characterized by structural simplicity, a bounded state space, and ease of implementation. The mathematical form of the map is given in Equation (1).   

(1) $$x_{n+1}=mod\left({mx}_{n},1\right)$$

where $x_{n}$ denotes the system state and *m* is the control parameter. When m>1, the system generates complex trajectories through stretching and folding. However, since this system contains only a single state variable and has a low dynamical dimension, there are still certain limitations regarding state ergodicity and complexity.

### 2.2. Construction of the Proposed Three-Dimensional Sine-Bilinear Fully Coupled Chaotic Map

To enhance the complexity and ergodicity of discrete chaotic systems, two additional state variables are introduced within the framework of the classical mod-1 map, thereby extending the original one-dimensional system to a three-dimensional one. In addition, a sinusoidal term is incorporated into the state equations to introduce periodic nonlinear perturbations, while linear coupling and bilinear cross-coupling terms are employed to strengthen the interactions among the state variables. Based on this design, a three-dimensional sine-bilinear fully coupled chaotic map (3D-SBFCM) is proposed, and its mathematical form is given in Equation (2).

(2) $$\left\{\begin{array}{c} x_{n+1}=\;\mathrm{mod}\;\left(mx_{n}+\alpha \sin\left(2\pi y_{n}\right)+\beta z_{n}+\gamma y_{n}z_{n},1\right)\\ y_{n+1}=\;\mathrm{mod}\;\left(my_{n}+\alpha \sin\left(2\pi z_{n}\right)+\beta x_{n+1}+\gamma z_{n}x_{n+1},1\right)\\ z_{n+1}=\;\mathrm{mod}\;\left(mz_{n}+\alpha \sin\left(2\pi x_{n+1}\right)+\beta y_{n+1}+\gamma x_{n+1}y_{n+1},1\right) \end{array}\right.$$

In this map, *m* controls the linear expansion strength and serves as a key factor in chaos generation; α determines the strength of sinusoidal modulation and enhances the irregularity and sensitivity of state evolution; β denotes the linear coupling strength; and γ denotes the bilinear cross-coupling strength. Owing to the synergistic effects of these nonlinear mechanisms, the system exhibits complex chaotic dynamics, thus laying the foundation for subsequent keystream generation.

### 2.3. Chaotic Analysis of the Proposed 3D-SBFCM

To verify the chaotic properties of the proposed 3D-SBFCM, phase portraits, Lyapunov exponents, permutation entropy, the 0-1 test, and the NIST SP 800-22 test are employed to perform a comprehensive evaluation in terms of trajectory distribution, sensitivity to initial conditions, time-series complexity, and statistical randomness.

#### 2.3.1. Phase Portrait

Phase portraits visually illustrate the distribution of system trajectories in state space and therefore serve as useful tools for evaluating ergodicity and attractor structure. If a system exhibits pronounced chaotic behavior, its trajectories are typically distributed more widely throughout the phase space, and its attractor structure tends to be more complex [30].

**Figure 1:**
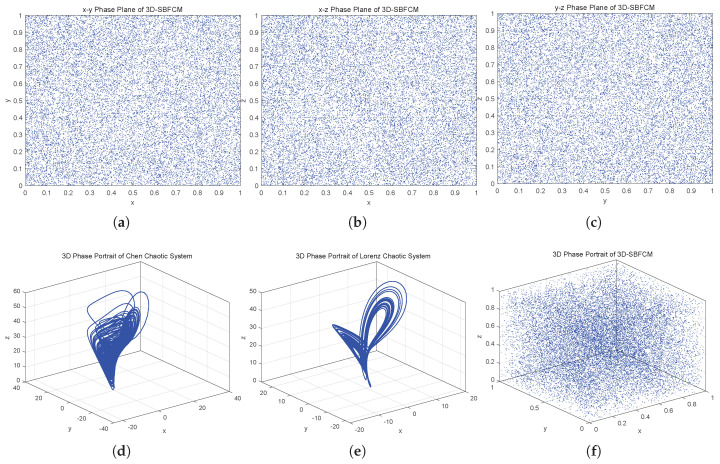
Phase portraits of chaotic systems. (**a**) x–y phase plane of the 3D-SBFCM system; (**b**) x–z phase plane of the 3D-SBFCM system; (**c**) y–z phase plane of the 3D-SBFCM system; (**d**) three-dimensional phase portrait of the Chen system; (**e**) three-dimensional phase portrait of the Lorenz system; and (**f**) three-dimensional phase portrait of the 3D-SBFCM system.

Figure 1 shows the two-dimensional phase portraits of the 3D-SBFCM and compares them with the three-dimensional phase portraits of the Chen and Lorenz systems. In the simulations, the parameters of the 3D-SBFCM were set as m=3.7, α=0.5, β=0.3, and γ=0.2, with initial conditions $x_{0}=0.1234$, $y_{0}=0.2345$, and $z_{0}=0.3456$.

As shown in Figure 1a–c, the state points of the 3D-SBFCM are relatively uniformly distributed over different projection planes, without obvious periodic clustering, indicating good ergodicity. As shown in Figure 1d–f, compared with the localized attractor trajectories of the Chen and Lorenz systems, the proposed 3D-SBFCM displays a more uniform distribution within the bounded unit state space.

#### 2.3.2. Lyapunov Exponents

The Lyapunov exponent (LE) provides a quantitative measure of a nonlinear system’s sensitivity to initial conditions. In general, chaotic systems have one positive LE, whereas hyperchaotic systems have at least two positive LEs [31].

**Figure 2:**
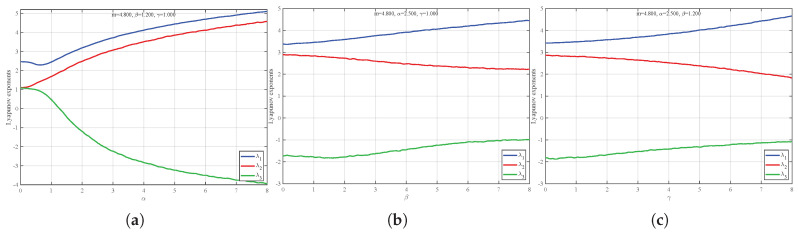

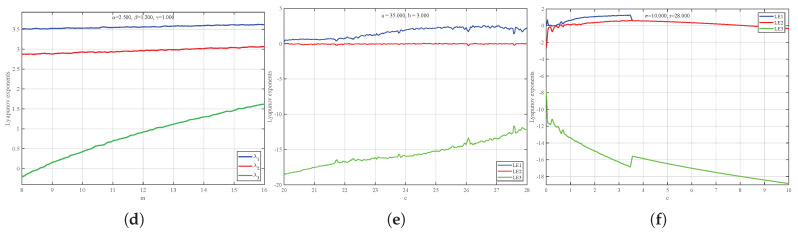
Lyapunov exponents of different chaotic systems. (**a**) 3D-SBFCM system with varying α; (**b**) 3D-SBFCM system with varying β; (**c**) 3D-SBFCM system with varying γ; (**d**) 3D-SBFCM system with varying *m*; (**e**) Chen system with varying *c*; and (**f**) Lorenz system with varying *c*.

Figure 2 shows the Lyapunov exponents of the 3D-SBFCM, the Chen system, and the Lorenz system. The results indicate that the proposed system exhibits two positive Lyapunov exponents in most of the tested parameter ranges. This confirms that the 3D-SBFCM exhibits stable hyperchaotic behavior and good parameter robustness. By contrast, the parameter intervals over which the Chen and Lorenz systems exhibit positive LEs are narrower and show stronger fluctuations, indicating that their chaotic dynamics are less stable under parameter variations than those of the 3D-SBFCM.

#### 2.3.3. Permutation Entropy

**Figure 3:**
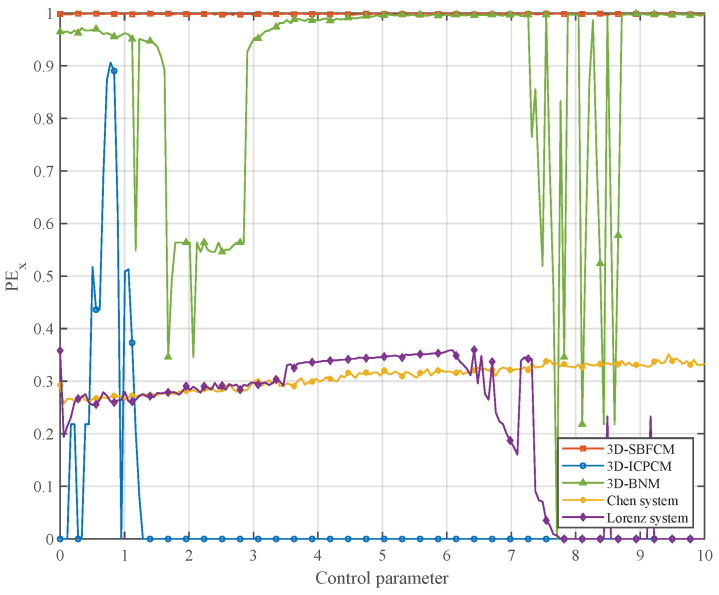
Permutation entropy of different chaotic systems.

Permutation entropy (PE) characterizes the complexity of a time series through the probability distribution of local ordering patterns. A larger PE value indicates greater randomness and temporal complexity in the sequence. The mathematical expression of PE is given in Equation (3).

(3) $$PE=-\frac{\sum_{l=1}^{K}p_{l}\ln p_{l}}{\ln(m!)}$$

where *m* is the embedding dimension, K=m! denotes the total number of possible permutation patterns, and $p_{l}$ is the probability of occurrence of the *l*-th permutation pattern. In this paper, we set the embedding dimension to m=4 and the delay to 1, and performed permutation entropy analysis on the 3D-SBFCM, 3D-ICPCM [32], 3D-BNM [33], Chen system, and Lorenz system. The results are shown in Figure 3.

Figure 3 shows that the 3D-SBFCM maintains relatively high and stable PE values over a wide range of parameter values, indicating strong temporal complexity in its output sequences. In contrast, 3D-ICPCM and 3D-BNM exhibit more obvious fluctuations in certain parameter intervals, which indicates that their time-series complexity is less stable under parameter variations. The Chen system exhibits a moderate PE level, whereas the Lorenz system shows relatively low PE values in certain parameter intervals, indicating relatively limited temporal complexity in these systems.

#### 2.3.4. 0-1 Test

The 0-1 test is used to determine whether a system exhibits chaotic behavior by constructing translation variables and examining the asymptotic growth of the mean square displacement. Its formulation is given in Equation (4).

(4) $$\left\{\begin{array}{c} p\left(n\right)=\sum_{i=1}^{n}\phi \left(i\right)\cos\left(ic\right)\\ q\left(n\right)=\sum_{i=1}^{n}\phi \left(i\right)\sin\left(ic\right)\\ M\left(n\right)=\frac{1}{\left(N-n\right)}\sum_{i=1}^{N-n}\left\{{\left[p\left(i+n\right)-p\left(i\right)\right]}^{2}+{\left[q\left(i+n\right)-q\left(i\right)\right]}^{2}\right\}\\ K_{c}=\mathrm{Corr}\left(\lambda ,a\right)=\frac{\mathrm{Cov}(\lambda ,a)}{\sqrt{\mathrm{Var}(\lambda )\mathrm{Var}(a)}}\\ \lambda =\left(1,2,\ldots ,n_{cut}\right)\\ a=\left(M\left(1\right),M\left(2\right),\ldots ,M\left(n_{cut}\right)\right)\\ K=\mathrm{median}\left(K_{c_{1}},K_{c_{2}},\ldots ,K_{c_{n}}\right) \end{array}\right.$$

Here, c∈(0,π) is a randomly selected parameter, and the statistic *K* is taken as the median of the results corresponding to multiple random values of *c*. When K≈1, the system is considered chaotic; when K≈0, the system is considered non-chaotic [34].

**Figure 4:**
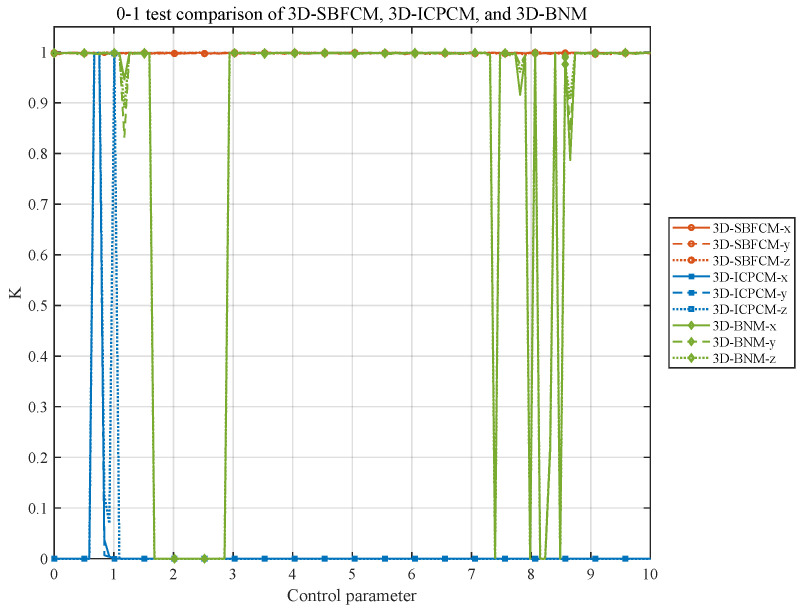
The 0-1 test results for different chaotic systems.

Figure 4 presents the 0-1 test results for the 3D-SBFCM, 3D-ICPCM, and 3D-BNM. It is clear that the K values corresponding to the three state variables of the 3D-SBFCM remain close to 1 throughout the tested parameter range, indicating that the proposed system can maintain stable chaotic behavior over a broad parameter interval.

#### 2.3.5. NIST SP 800-22 Test

To further verify the pseudorandomness of the output sequence generated by the 3D-SBFCM, the NIST SP 800-22 test suite [35] is employed for evaluation. For the test, the initial states were set to $x_{0}=0.1234$, $y_{0}=0.2345$, and $z_{0}=0.3456$. The results are listed in Table 1. The NIST SP 800-22 suite contains 15 test categories. Among them, the Random Excursion Test and the Random Excursion Variant Test include multiple state-dependent subtests. For compact presentation, Table 1 reports the minimum *p*-value among all valid state-dependent subtests for these two categories. All reported *p*-values are greater than 0.01, and all corresponding outcomes are marked as “Pass”. These results demonstrate that the proposed 3D-SBFCM is capable of generating sequences with good statistical randomness, making it suitable for image encryption applications.

## 3. Encryption and Decryption Procedures

**Figure 5:**
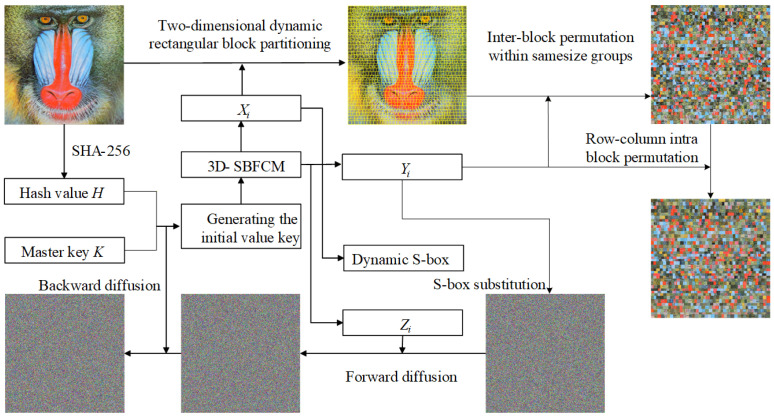
Flowchart of the proposed color image encryption algorithm.

The purpose of image encryption is to weaken the spatial correlation and statistical regularity of plaintext images while maintaining strict reversibility. In block-based encryption algorithms, the partitioning strategy is crucial for confusion performance and for reducing residual structural information in the ciphertext. Although fixed-size partitioning is simple to implement, its regular block boundaries may retain certain spatial structures, thereby increasing the risk of visual information leakage. To address this issue, a color image encryption algorithm based on the proposed 3D-SBFCM is developed. The algorithm employs a hierarchical framework that incorporates plaintext-related key modulation, dynamic rectangular partitioning, inter-block permutation, intra-block permutation, dynamic S-box substitution, and forward–backward cross-channel diffusion. The overall framework of the proposed algorithm is illustrated in Figure 5.

### 3.1. Key Generation and Chaotic Sequence Construction

#### 3.1.1. Master Key

The master key is defined as

(5) $$K=\left\{x_{0},y_{0},z_{0},m,\alpha ,\beta ,\gamma ,\eta_{1},\eta_{2},\eta_{3}\right\},$$

where $\left(x_{0},y_{0},z_{0}\right)$ denote the initial states of the 3D-SBFCM; *m*, α, β, and γ denote the control parameters; and $\eta_{1}$, $\eta_{2}$, and $\eta_{3}\in \left[0,255\right]$ are auxiliary seeds.

#### 3.1.2. Plaintext-Related Initialization

Let the input color image be denoted by

(6) $$I\in Z_{256}^{M\times N\times 3},$$

where *M* and *N* denote the image dimensions, and $Z_{256}$ denotes the gray-value domain [0,255].

The image is serialized in row-major order with the channel order R-G-B, and a SHA-256 digest is computed from the resulting byte stream. The digest is denoted by

(7) $$H=\left\{h_{1},h_{2},\ldots ,h_{32}\right\},h_{i}\in \left[0,255\right].$$

To enable the receiver to regenerate the same chaotic control sequences during decryption, the digest *H* is transmitted together with the ciphertext as public session synchronization information. Since *H* is not secret, it is not regarded as part of the secret key and is not included in the key-space calculation.

Although an attacker may obtain or modify *H*, the valid chaotic sequences and diffusion feedback values are generated jointly from *H* and the secret master key. Therefore, modifying *H* can only affect the sequences generated at the receiver side, but cannot enable the attacker to predict, control, or reconstruct the valid encryption sequences without the secret master key. If a modified *H* is used in decryption, the generated sequences will be inconsistent with those used during encryption, resulting in decryption failure. Thus, such manipulation mainly leads to denial of decryption rather than plaintext recovery, key recovery, or a compromise of confidentiality.

The digest *H* and the auxiliary seeds $\eta_{1}$, $\eta_{2}$, and $\eta_{3}$ are further used to modulate the initial states of the 3D-SBFCM, producing the updated states $\left(x_{0}^{\prime },y_{0}^{\prime },z_{0}^{\prime }\right)$:

(8) $$\left\{\begin{array}{cc} x_{0}^{\prime } & =\mathrm{mod}\left(x_{0}+\frac{\left[\left(\sum_{i=1}^{10}h_{i}\right)\;\mathrm{mod}\;256\right]⊕\eta_{1}}{256},1\right)\\ y_{0}^{\prime } & =\mathrm{mod}\left(y_{0}+\frac{\left[\left(\sum_{i=11}^{21}h_{i}\right)\;\mathrm{mod}\;256\right]⊕\eta_{2}}{256},1\right)\\ z_{0}^{\prime } & =\mathrm{mod}\left(z_{0}+\frac{\left[\left(\sum_{i=22}^{32}h_{i}\right)\;\mathrm{mod}\;256\right]⊕\eta_{3}}{256},1\right) \end{array}\right.$$

The 32-byte digest is partitioned into three segments of lengths 10, 11, and 11 for state modulation. In this way, the master key is coupled with the plaintext image, making the generated control sequences highly sensitive to plaintext variations.

The 3D-SBFCM is then iterated using $\left(x_{0}^{\prime },y_{0}^{\prime },z_{0}^{\prime }\right)$ and the parameters *m*, α, β, and γ. After discarding the first $N_{0}$ transient values, three effective chaotic sequences are obtained:

(9) $$X=\left\{x_{1},x_{2},\ldots ,x_{T}\right\},\;Y=\left\{y_{1},y_{2},\ldots ,y_{T}\right\},\;Z=\left\{z_{1},z_{2},\ldots ,z_{T}\right\}.$$

where *T* denotes the total sequence length required by all encryption stages.

For integer-domain operations, the continuous sequences are quantized as:

(10) $$\left\{\begin{array}{cc} X_{i} & =\;\mathrm{mod}\;\left(\left\lfloor x_{i}\times 10^{14}\right\rfloor ,256\right)\\ Y_{i} & =\;\mathrm{mod}\;\left(\left\lfloor y_{i}\times 10^{14}\right\rfloor ,256\right)\\ Z_{i} & =\;\mathrm{mod}\;\left(\left\lfloor z_{i}\times 10^{14}\right\rfloor ,256\right) \end{array}\right.$$

Specifically, the quantized sequence $\left\{X_{i}\right\}$ is used for dynamic partitioning and basic S-box construction, $\left\{Y_{i}\right\}$ is used for permutation control, and $\left\{Z_{i}\right\}$ is used for diffusion. To avoid reusing the same control variables in different modules, all quantized sequences are consumed sequentially by independent read pointers, which are advanced after each module is completed.

### 3.2. Two-Dimensional Dynamic Rectangular Partitioning

To reduce the structural regularity introduced by fixed-size blocking, a two-dimensional dynamic rectangular partitioning strategy is employed. In conventional fixed-size partitioning, block boundaries are located at regular row and column intervals, forming a periodic grid-like structure. Such a regular partitioning pattern may still leave residual boundary traces after block-level scrambling. In contrast, the proposed method first divides the image into horizontal strips with unequal heights and then partitions each strip into rectangular blocks with variable widths using chaotic sequences. Consequently, the boundary positions become non-periodic and dynamically controlled, which reduces the residual grid-like regularity caused by fixed block partitioning. Since only pixel positions are rearranged at this stage, reversibility is strictly preserved. For color images, identical block boundaries are used for the R, G, and B channels to maintain spatial consistency across channels.

To ensure that the remaining rows or columns can always be partitioned in a feasible manner, a residual feasibility condition is introduced. For a remaining length *R*, lower bound $d_{\min}$, and upper bound $d_{\max}$, the residual length is considered feasible if R=0, or if there exists a positive integer *n*, representing the number of remaining segments, such that $nd_{\min}\leq R\leq nd_{\max}$.

Equivalently, the feasibility condition can be written as

(11) $$\left\lceil \frac{R}{d_{\max}}\right\rceil \leq \left\lfloor \frac{R}{d_{\min}}\right\rfloor .$$

This condition is used in the following dynamic height and width generation procedures to guarantee that the image can be completely partitioned without omission or overlap.

#### 3.2.1. Dynamic Horizontal Strip Partitioning

Let $H_{\min}$ and $H_{\max}$ denote the minimum and maximum strip heights, respectively, satisfying

(12) $$1\leq H_{\min}\leq H_{\max}\leq M.$$

The selected parameters should satisfy the feasibility condition for the whole image height, namely

(13) $$\left\lceil \frac{M}{H_{\max}}\right\rceil \leq \left\lfloor \frac{M}{H_{\min}}\right\rfloor .$$

Let $R_{h}^{\left(u\right)}$ denote the number of remaining unpartitioned rows after the first *u* strips have been determined. The initialization is given by   

(14) $$R_{h}^{\left(0\right)}=M.$$

If the image is finally divided into *U* strips and $h_{u}$ denotes the height of the *u*-th strip, then

(15) $$H_{\min}\leq h_{u}\leq H_{\max},\;u=1,2,\ldots ,U.$$

and

(16) $$\sum_{u=1}^{U}h_{u}=M.$$

The candidate strip height is first generated from the quantized chaotic sequence as

(17) $${\hat{h}}_{u}=H_{\min}+\left(X_{ptr_{X}}\;\mathrm{mod}\;\left(H_{\max}-H_{\min}+1\right)\right),$$

where $ptr_{X}$ denotes the current reading pointer of $\left\{X_{i}\right\}$. After each generation, the pointer is updated as $ptr_{X}\leftarrow ptr_{X}+1$. Here, ${\hat{h}}_{u}$ is only a preliminary candidate rather than the final height of the current strip. The actual strip height $h_{u}$ is determined by checking whether the remaining number of rows can still be partitioned into valid strips.

To this end, we define a height feasibility predicate $\Phi_{H}\left(R\right)$. For a residual height *R*, $\Phi_{H}\left(R\right)$ returns true if *R* can be further divided into one or more valid strips whose heights lie within $\left[H_{\min},H_{\max}\right]$. Specifically,

(18) $$\Phi_{H}\left(R\right)=\mathrm{true}⟺\left\lceil \frac{R}{H_{\max}}\right\rceil \leq \left\lfloor \frac{R}{H_{\min}}\right\rfloor .$$

If the residual height after selecting ${\hat{h}}_{u}$ does not satisfy this condition, the candidate height is cyclically adjusted within the allowable interval until the residual height becomes feasible. The residual-constrained height determination procedure is summarized in Algorithm 1.
**Algorithm 1** Residual-constrained determination of strip height.**Input:** Residual height $R_{h}^{\left(u-1\right)}$, height bounds $H_{\min}$ and $H_{\max}$, candidate height ${\hat{h}}_{u}$**Output:** Selected height $h_{u}$, updated residual height $R_{h}^{\left(u\right)}$ 1:**if** $H_{\min}\leq R_{h}^{\left(u-1\right)}\leq H_{\max}$ **then** 2:      $h_{u}\leftarrow R_{h}^{\left(u-1\right)}$ 3:**else** 4:      $h_{u}\leftarrow {\hat{h}}_{u}$ 5:      $R_{\mathrm{temp}}\leftarrow R_{h}^{\left(u-1\right)}-h_{u}$ 6:      **if** $\Phi_{H}\left(R_{\mathrm{temp}}\right)=\mathrm{false}$ **then** 7:            Δ←1 8:            **while** $\Delta \leq H_{\max}-H_{\min}$ **do** 9:               $h_{u}\leftarrow H_{\min}+\left({\hat{h}}_{u}-H_{\min}+\Delta \right)\;\mathrm{mod}\;\left(H_{\max}-H_{\min}+1\right)$10:               $R_{\mathrm{temp}}\leftarrow R_{h}^{\left(u-1\right)}-h_{u}$11:               **if** $\Phi_{H}\left(R_{\mathrm{temp}}\right)=\mathrm{true}$ **then**12:                     **break**13:               **end if**14:               Δ←Δ+115:            **end while**16:      **end if**17:**end if**18:$R_{h}^{\left(u\right)}\leftarrow R_{h}^{\left(u-1\right)}-h_{u}$19:**return** $h_{u}$, $R_{h}^{\left(u\right)}$

After strip partitioning is completed, the starting and ending row indices of the *u*-th strip are given by

(19) $$\left\{\begin{array}{cc} r_{u}^{\left(s\right)} & =1+\sum_{i=1}^{u-1}h_{i}\\ r_{u}^{\left(e\right)} & =\sum_{i=1}^{u}h_{i} \end{array}\right.$$

Therefore, the *u*-th strip can be expressed as

(20) $$S_{u}=I\left[r_{u}^{\left(s\right)}:r_{u}^{\left(e\right)},1:N,:\right].$$

#### 3.2.2. Dynamic Column-Wise Partitioning Within Each Strip

After all dynamic strips are obtained, each strip is further partitioned along the column direction. Let $W_{\min}$ and $W_{\max}$ denote the minimum and maximum block widths, respectively, satisfying

(21) $$1\leq W_{\min}\leq W_{\max}\leq N.$$

Similarly, the selected width parameters should satisfy

(22) $$\left\lceil \frac{N}{W_{\max}}\right\rceil \leq \left\lfloor \frac{N}{W_{\min}}\right\rfloor .$$

For the *u*-th strip $S_{u}$, let $R_{w,u}^{\left(v\right)}$ denote the number of remaining unpartitioned columns after the first *v* blocks have been determined. The initialization is

(23) $$R_{w,u}^{\left(0\right)}=N.$$

Assume that the *u*-th strip is finally divided into $T_{u}$ rectangular blocks, and let $w_{u,v}$ denote the width of the *v*-th block. Then,

(24) $$W_{\min}\leq w_{u,v}\leq W_{\max},\;v=1,2,\ldots ,T_{u}.$$

and

(25) $$\sum_{v=1}^{T_{u}}w_{u,v}=N.$$

Within each strip, the subsequent control values of the quantized sequence $\left\{X_{i}\right\}$ are used to generate the candidate block width:

(26) $${\hat{w}}_{u,v}=W_{\min}+\left(X_{ptr_{X}}\;\mathrm{mod}\;\left(W_{\max}-W_{\min}+1\right)\right).$$

After each generation, the pointer is updated as $ptr_{X}\leftarrow ptr_{X}+1$. Here, ${\hat{w}}_{u,v}$ is only a preliminary candidate rather than the final width of the current block. The actual block width $w_{u,v}$ is determined by checking whether the remaining number of columns can still be partitioned into valid blocks.

Similar to the strip-height determination, we define a width feasibility predicate $\Phi_{W}\left(R\right)$. For a residual width *R*, $\Phi_{W}\left(R\right)$ returns true if R=0, or if *R* can be further divided into one or more valid blocks whose widths lie within $\left[W_{\min},W_{\max}\right]$. Specifically,

(27) $$\Phi_{W}\left(R\right)=\mathrm{true}⟺\left\lceil \frac{R}{W_{\max}}\right\rceil \leq \left\lfloor \frac{R}{W_{\min}}\right\rfloor .$$

If the residual width after selecting ${\hat{w}}_{u,v}$ does not satisfy this condition, the candidate width is cyclically adjusted within the allowable interval until the residual width becomes feasible. The residual-constrained width determination procedure is summarized in Algorithm 2.
**Algorithm 2** Residual-constrained determination of block width.**Input:** Residual width $R_{w,u}^{\left(v-1\right)}$, width bounds $W_{\min}$ and $W_{\max}$, candidate width ${\hat{w}}_{u,v}$**Output:** Selected width $w_{u,v}$, updated residual width $R_{w,u}^{\left(v\right)}$ 1:**if** $W_{\min}\leq R_{w,u}^{\left(v-1\right)}\leq W_{\max}$ **then** 2:      $w_{u,v}\leftarrow R_{w,u}^{\left(v-1\right)}$ 3:**else** 4:      $w_{u,v}\leftarrow {\hat{w}}_{u,v}$ 5:      $R_{\mathrm{temp}}\leftarrow R_{w,u}^{\left(v-1\right)}-w_{u,v}$ 6:      **if** $\Phi_{W}\left(R_{\mathrm{temp}}\right)=\mathrm{false}$ **then** 7:            Δ←1 8:            **while** $\Delta \leq W_{\max}-W_{\min}$ **do** 9:                 $w_{u,v}\leftarrow W_{\min}+\left({\hat{w}}_{u,v}-W_{\min}+\Delta \right)\;\mathrm{mod}\;\left(W_{\max}-W_{\min}+1\right)$10:                 $R_{\mathrm{temp}}\leftarrow R_{w,u}^{\left(v-1\right)}-w_{u,v}$11:                 **if** $\Phi_{W}\left(R_{\mathrm{temp}}\right)=\mathrm{true}$ **then**12:                       **break**13:                 **end if**14:                 Δ←Δ+115:            **end while**16:      **end if**17:**end if**18:$R_{w,u}^{\left(v\right)}\leftarrow R_{w,u}^{\left(v-1\right)}-w_{u,v}$19:**return** $w_{u,v}$, $R_{w,u}^{\left(v\right)}$

After determining the block widths, the starting and ending column indices of the *v*-th block in the *u*-th strip are given by

(28) $$\left\{\begin{array}{cc} c_{u,v}^{\left(s\right)} & =1+\sum_{j=1}^{v-1}w_{u,j}\\ c_{u,v}^{\left(e\right)} & =\sum_{j=1}^{v}w_{u,j} \end{array}\right.$$

Accordingly, the *v*-th block in the *u*-th strip is defined as

(29) $$B_{u,v}=I\left[r_{u}^{\left(s\right)}:r_{u}^{\left(e\right)},\;c_{u,v}^{\left(s\right)}:c_{u,v}^{\left(e\right)},\;:\right].$$

Repeating the above procedure for all strips yields the complete set of two-dimensional dynamic rectangular blocks:

(30) $$B=\left\{B_{u,v}\;|\;1\leq u\leq U,\;1\leq v\leq T_{u}\right\}.$$

Let *Q* denote the total number of generated blocks. Then,

(31) $$Q=\sum_{u=1}^{U}T_{u}.$$

The size of each block $B_{u,v}$ is

(32) $$\mathrm{size}\left(B_{u,v}\right)=h_{u}\times w_{u,v}.$$

According to the above construction, the image is partitioned into a set of rectangular blocks with irregular boundaries and variable sizes. These blocks are non-overlapping and collectively cover the entire image domain without omission. Therefore, the proposed partitioning scheme provides a reversible structural basis for the subsequent scrambling and reconstruction stages.

### 3.3. Inter-Block Permutation Based on Same-Size Grouping

After the two-dimensional dynamic rectangular partitioning, the image is decomposed into multiple rectangular blocks of different sizes. If blocks with different sizes are directly exchanged, boundary mismatch will occur during reconstruction. Therefore, a same-size grouping and intra-group permutation strategy is adopted for inter-block permutation.

Let the block set be denoted by

(33) $$B=\{B_{1},B_{2},\ldots ,B_{Q}\}.$$

The size of the *k*-th block is denoted by

(34) $$\mathrm{size}\left(B_{k}\right)=\left(h_{k},w_{k}\right),$$

where size(·) denotes the height–width pair of a block.

According to block size, all blocks are divided into *P* same-size groups:

(35) $$G=\{G_{1},G_{2},\ldots ,G_{P}\}.$$

Assume that the *p*-th group contains $n_{p}$ blocks. Then,

(36) $$\left\{\begin{array}{c} G_{p}=\left\{B_{p,1},B_{p,2},\ldots ,B_{p,n_{p}}\right\}\\ \sum_{p=1}^{P}n_{p}=Q \end{array}\right.\;p=1,2,\ldots ,P$$

For each group $G_{p}$, a control vector of length $n_{p}$ is extracted from the quantized chaotic sequence $\left\{Y_{i}\right\}$:

(37) $$s_{p}=\left\{s_{p,1},s_{p,2},\ldots ,s_{p,n_{p}}\right\}.$$

The control vector $s_{p}$ is sorted in ascending order to obtain the permutation index sequence.

(38) $$\pi_{p}=\mathrm{sortindex}\left(s_{p}\right).$$

The blocks within the *p*-th group are then rearranged according to $\pi_{p}$

(39) $$B_{p,i}^{\prime }=B_{p,\pi_{p}\left(i\right)},\;i=1,2,\ldots ,n_{p}$$

If $n_{p}=1$, the corresponding block remains unchanged in the inter-block permutation stage. Because this operation is carried out only among blocks with identical sizes, block boundary compatibility is preserved. Meanwhile, the global spatial layout of image regions is disrupted. During decryption, the original block positions can be exactly restored by applying the inverse permutation $\pi_{p}^{-1}$.

### 3.4. Two-Dimensional Intra-Block Permutation of Dynamic Rectangular Blocks

Although inter-block permutation changes the macroscopic positions of image regions, the local adjacency of pixels inside each block is still partially preserved. To further reduce local correlation, a two-dimensional intra-block permutation is performed within each block.

Let the *k*-th block after inter-block permutation be denoted by $B_{k}^{\prime }$, whose size is $h_{k}\times w_{k}$. From the chaotic sequence $\left\{Y_{i}\right\}$, two control vectors are further extracted:

(40) $$\left\{\begin{array}{c} a_{k}=a_{k,1},a_{k,2},\ldots ,a_{k,h_{k}}\\ b_{k}=b_{k,1},b_{k,2},\ldots ,b_{k,w_{k}} \end{array}\right.$$

By sorting $a_{k}$ and $b_{k}$ in ascending order, the row and column permutation sequences are obtained as

(41) $$\left\{\begin{array}{cc} \alpha_{k} & =\mathrm{sortindex}(a_{k})\\ \beta_{k} & =\mathrm{sortindex}(b_{k}) \end{array}\right.$$

The rows of $B_{k}^{\prime }$ are first rearranged according to $\alpha_{k}$, and the columns are then rearranged according to $\beta_{k}$. The resulting block after the intra-block permutation is denoted by

(42) $${\tilde{B}}_{k}=\beta_{k}\left(\alpha_{k}\left(B_{k}^{\prime }\right)\right).$$

The same row and column permutation sequences are shared by the R, G, and B channels so that inter-channel spatial consistency is preserved. After this stage, both the global positions of blocks and the local structures inside the blocks have been significantly disturbed, thereby preparing the image data for the subsequent value-domain encryption process.

**Figure 6:**
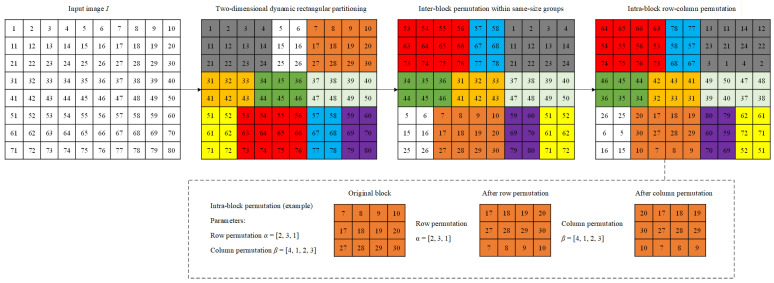
Example of inter-block and intra-block permutation based on dynamic rectangular partitioning.

An illustrative example of the inter-block and intra-block permutation process is shown in Figure 6.

### 3.5. Dynamic S-Box Substitution and Forward–Backward Cross-Channel Diffusion

The above scrambling operations effectively destroy the spatial correlation of the image, but the pixel intensities themselves remain largely unchanged. Therefore, a value-domain encryption stage is further introduced to enhance resistance against statistical and differential attacks. Specifically, dynamic S-box substitution is first employed to achieve nonlinear pixel transformation, and forward–backward cross-channel diffusion is then performed to propagate local perturbations over the entire image.

#### 3.5.1. Dynamic S-Box Construction

Let the permuted color image obtained after the previous stages be denoted by

(43) $$P=\{P_{R},P_{G},P_{B}\}$$

Using a unified scanning rule, the three channels are serialized into one-dimensional sequences of length L=M×N:

(44) $${\left\{p_{R}\left(t\right)\right\}}_{t=1}^{L},{\left\{p_{G}\left(t\right)\right\}}_{t=1}^{L},{\left\{p_{B}\left(t\right)\right\}}_{t=1}^{L}$$

To construct the basic S-box, the first 256 mutually distinct integers are extracted from the quantized chaotic sequence $\left\{X_{i}\right\}$, forming the set

(45) $$V_{s}=\left\{V_{s}\left(0\right),V_{s}\left(1\right),\ldots ,V_{s}\left(255\right)\right\}.$$

If fewer than 256 distinct elements are obtained during the scanning process, the absent elements are supplemented in natural order until a complete permutation of {0,1,…,255} is formed. By sorting $V_{s}$ in ascending order and recording the original indices, a permutation sequence is obtained:

(46) Π={π(0),π(1),…,π(255)}.

Accordingly, the basic S-box is defined as

(47) $$S_{0}\left(i\right)=\pi \left(i\right),\;i\in \left\{0,1,\ldots ,255\right\}$$

and its inverse mapping is denoted by $S_{0}^{-1}$.

To introduce position dependency, for channel χ∈{R,G,B} and pixel position *t*, the shift quantity $\mu_{\chi }\left(t\right)$ and mask quantity $v_{\chi }\left(t\right)$ are defined as

(48) $$\left\{\begin{array}{cc} \mu_{\chi }\left(t\right) & =X_{\chi }\left(t\right)\;\mathrm{mod}\;256\\ v_{\chi }\left(t\right) & =Y_{\chi }\left(t\right)\;\mathrm{mod}\;256 \end{array}\right.$$

where $X_{\chi }\left(t\right)$ and $Y_{\chi }\left(t\right)$ denote the corresponding control mappings, and the next available control values are sequentially read from the quantized chaotic sequences $\left\{X_{i}\right\}$ and $\left\{Y_{i}\right\}$, respectively, for channel χ at position *t*.

Then, the dynamic S-box substitution result of channel χ at position *t* is given by

(49) $$u_{\chi }\left(t\right)=S_{0}\left(\left(p_{\chi }\left(t\right)+\mu_{\chi }\left(t\right)\right)\;\mathrm{mod}\;256\right)⊕v_{\chi }\left(t\right)$$

After dynamic S-box substitution, three intermediate sequences are obtained:

(50) $${\left\{u_{R}\left(t\right)\right\}}_{t=1}^{L},{\left\{u_{G}\left(t\right)\right\}}_{t=1}^{L},{\left\{u_{B}\left(t\right)\right\}}_{t=1}^{L}$$

#### 3.5.2. Forward Cross-Channel Diffusion

The keystreams required for forward and backward diffusion are all derived from the quantized chaotic sequence $\left\{Z_{i}\right\}$. To avoid reusing the same random segment in both diffusion stages, $\left\{Z_{i}\right\}$ is sequentially divided into six non-overlapping subsequences:

(51) $$k_{R}^{f}\left(t\right)=Z_{6t-5},\;k_{G}^{f}\left(t\right)=Z_{6t-4},\;k_{B}^{f}\left(t\right)=Z_{6t-3}$$

(52) $$k_{R}^{b}\left(t\right)=Z_{6t-2},\;k_{G}^{b}\left(t\right)=Z_{6t-1},\;k_{B}^{b}\left(t\right)=Z_{6t}$$

where Equation (51) corresponds to the forward diffusion keystreams, and Equation (52) corresponds to the backward diffusion keystreams.

To enhance the joint sensitivity of the diffusion process to both the master key and the plaintext content, the auxiliary seeds and the digest *H* are used to calculate the initial feedback vectors $q_{R}^{\left(0\right)}$, $q_{G}^{\left(0\right)}$, and $q_{B}^{\left(0\right)}$ of the forward diffusion, which are defined as

(53) $$\left\{\begin{array}{cc} q_{R}\left(0\right) & =\eta_{1}⊕h_{1}⊕h_{2}⊕h_{3}⊕h_{4}\\ q_{G}\left(0\right) & =\eta_{2}⊕h_{5}⊕h_{6}⊕h_{7}⊕h_{8}\\ q_{B}\left(0\right) & =\eta_{3}⊕h_{9}⊕h_{10}⊕h_{11}⊕h_{12} \end{array}\right.$$

Let the intermediate cipher sequences after forward diffusion be denoted by

(54) $${\left\{q_{R}\left(t\right)\right\}}_{t=1}^{L},{\left\{q_{G}\left(t\right)\right\}}_{t=1}^{L},{\left\{q_{B}\left(t\right)\right\}}_{t=1}^{L}$$

Then, the forward cross-channel diffusion is defined as

(55) $$\left\{\begin{array}{cc} q_{R}\left(t\right) & =u_{R}\left(t\right)⊕k_{R}^{f}\left(t\right)⊕q_{R}\left(t-1\right)⊕q_{B}\left(t-1\right)\\ q_{G}\left(t\right) & =u_{G}\left(t\right)⊕k_{G}^{f}\left(t\right)⊕q_{G}\left(t-1\right)⊕q_{R}\left(t\right)\\ q_{B}\left(t\right) & =u_{B}\left(t\right)⊕k_{B}^{f}\left(t\right)⊕q_{B}\left(t-1\right)⊕q_{G}\left(t\right) \end{array}\;t=1,2,\ldots ,L\right.$$

This diffusion structure establishes progressive coupling among the three channels, thereby increasing the propagation range of a local perturbation across the whole image.

#### 3.5.3. Backward Cross-Channel Diffusion

If only forward diffusion is employed, perturbations mainly propagate from the beginning to the end of the sequence, and the feedback from the latter part to the former part remains limited. To further enhance global coupling, a backward diffusion process from the end to the beginning is introduced.

Let $c_{R}\left(L+1\right)$, $c_{G}\left(L+1\right)$, and $c_{B}\left(L+1\right)$ denote the terminal feedback values of backward diffusion, defined as

(56) $$\left\{\begin{array}{cc} c_{R}\left(L+1\right) & =\eta_{1}⊕h_{13}⊕h_{14}⊕h_{15}⊕h_{16}\\ c_{G}\left(L+1\right) & =\eta_{2}⊕h_{17}⊕h_{18}⊕h_{19}⊕h_{20}\\ c_{B}\left(L+1\right) & =\eta_{3}⊕h_{21}⊕h_{22}⊕h_{23}⊕h_{24} \end{array}\right.$$

Let the final cipher sequences be denoted by

(57) $${\left\{c_{R}\left(t\right)\right\}}_{t=1}^{L},{\left\{c_{G}\left(t\right)\right\}}_{t=1}^{L},{\left\{c_{B}\left(t\right)\right\}}_{t=1}^{L}$$

The backward diffusion is defined as

(58) $$\left\{\begin{array}{cc} c_{B}\left(t\right) & =q_{B}\left(t\right)⊕k_{B}^{b}\left(t\right)⊕c_{B}\left(t+1\right)⊕c_{R}\left(t+1\right)\\ c_{G}\left(t\right) & =q_{G}\left(t\right)⊕k_{G}^{b}\left(t\right)⊕c_{G}\left(t+1\right)⊕c_{B}\left(t\right)\\ c_{R}\left(t\right) & =q_{R}\left(t\right)⊕k_{R}^{b}\left(t\right)⊕c_{R}\left(t+1\right)⊕c_{G}\left(t\right) \end{array}\;t=L,L-1,\ldots ,1\right.$$

By introducing reverse-direction feedback, perturbations generated in the tail part of the sequence can also affect the front part, which further strengthens the global diffusion capability of the proposed algorithm.

### 3.6. Decryption Procedure

The decryption procedure is the exact inverse of the encryption procedure. Let the input cipher image be denoted by

(59) $$C=\{C_{R},C_{G},C_{B}\}$$

Using the same scanning rule as that adopted in the encryption stage, the three cipher channels are serialized into one-dimensional sequences of length *L*:

(60) $${\left\{c_{R}\left(t\right)\right\}}_{t=1}^{L},{\left\{c_{G}\left(t\right)\right\}}_{t=1}^{L},{\left\{c_{B}\left(t\right)\right\}}_{t=1}^{L}$$

The backward diffusion defined in Equation (58) is first inverted, with the terminal feedback values initialized according to Equation (56), to recover the intermediate sequences after forward diffusion:

(61) $$\left\{\begin{array}{cc} q_{B}\left(t\right) & =c_{B}\left(t\right)⊕k_{B}^{b}\left(t\right)⊕c_{B}\left(t+1\right)⊕c_{R}\left(t+1\right)\\ q_{G}\left(t\right) & =c_{G}\left(t\right)⊕k_{G}^{b}\left(t\right)⊕c_{G}\left(t+1\right)⊕c_{B}\left(t\right)\\ q_{R}\left(t\right) & =c_{R}\left(t\right)⊕k_{R}^{b}\left(t\right)⊕c_{R}\left(t+1\right)⊕c_{G}\left(t\right) \end{array}\;t=L,L-1,\ldots ,1\right.$$

Subsequently, the forward cross-channel diffusion defined in Equation (55) is inverted, with the initial feedback values initialized according to Equation (53), to recover the sequences after dynamic S-box substitution:

(62) $$\left\{\begin{array}{cc} u_{R}\left(t\right) & =q_{R}\left(t\right)⊕k_{R}^{f}\left(t\right)⊕q_{R}\left(t-1\right)⊕q_{B}\left(t-1\right)\\ u_{G}\left(t\right) & =q_{G}\left(t\right)⊕k_{G}^{f}\left(t\right)⊕q_{G}\left(t-1\right)⊕q_{R}\left(t\right)\\ u_{B}\left(t\right) & =q_{B}\left(t\right)⊕k_{B}^{f}\left(t\right)⊕q_{B}\left(t-1\right)⊕q_{G}\left(t\right) \end{array}\;t=1,2,\ldots ,L\right.$$

Next, inverse dynamic S-box mapping is performed to recover the permuted pixel values:

(63) $$p_{\chi }\left(t\right)=\left[S_{0}^{-1}\left(u_{\chi }\left(t\right)⊕v_{\chi }\left(t\right)\right)-\mu_{\chi }\left(t\right)\right]\;\mathrm{mod}\;256$$

The recovered permuted sequences are then reorganized into a block set according to the regenerated dynamic rectangular boundaries. Let $\alpha_{k}^{-1}$ and $\beta_{k}^{-1}$ denote the inverse row and column permutations of the *k*-th block, respectively. Then, the inverse intra-block permutation is expressed as

(64) $$B_{k}^{\prime }=\alpha_{k}^{-1}\left(\beta_{k}^{-1}\left({\tilde{B}}_{k}\right)\right)$$

After that, the inverse permutation $\pi_{p}^{-1}$ is applied to each same-size block group to restore the original block positions. Finally, the restored R, G, and B channels are merged according to the regenerated dynamic block boundaries to reconstruct the original plaintext image *I*.

## 4. Security Analysis and Performance Evaluation

Unless otherwise specified, all experiments were conducted using MATLAB R2024a on a 64-bit Windows 10 Professional platform equipped with an Intel(R) Core(TM) i5-14600KF CPU @ 3.50 GHz, 32.0 GB RAM, and an NVIDIA GeForce RTX 5070 GPU with 12 GB video memory. No GPU acceleration or parallel computing toolbox was used in the reported experiments. The real-valued chaotic computations were performed using IEEE-754 double-precision floating-point arithmetic.

This section presents a systematic evaluation of the proposed 3D-SBFCM-based color image encryption algorithm with dynamic rectangular partitioning, dynamic S-box substitution, and forward-backward cross-channel diffusion. The assessment focuses on its security and practical robustness, including its ability to conceal plaintext information, resist common attacks, and maintain reliable performance under interference. Detailed analyses and experimental results are provided in the following sections.

### 4.1. Key Space Analysis

The secret key of the proposed algorithm consists of seven real-valued secret parameters, namely $x_{0}$, $y_{0}$, $z_{0}$, *m*, α, β, and γ, together with three auxiliary 8-bit seed parameters $\eta_{1}$, $\eta_{2}$, and $\eta_{3}$. Assuming that each real-valued secret parameter has an effective range of approximately one and is represented with a precision of $10^{-14}$, these seven parameters provide approximately $10^{98}$ possible combinations. In addition, the three 8-bit seed parameters contribute $256^{3}$ additional possibilities. Therefore, the total key space can be estimated as $10^{98}\times 256^{3}\approx 2^{350}$. Since this value is much larger than the commonly accepted threshold of $2^{100}$ for resisting brute-force attacks, the proposed algorithm provides a sufficiently large key space to resist brute-force attacks in practical image encryption applications.

It should be noted that the above key-space estimation depends on the numerical precision adopted in the implementation. Since the simulations in this work were performed using IEEE-754 double-precision floating-point arithmetic, the precision level of $10^{-14}$ is consistent with the distinguishable numerical resolution for the considered parameter ranges. However, finite-precision representation may narrow the effective key space in practical implementations, particularly when single-precision or fixed-point arithmetic is adopted. Therefore, the estimated key space of approximately $2^{350}$ should be regarded as a theoretical estimate under the adopted double-precision simulation environment, and the same numerical representation, rounding rule, and iteration procedure should be used in both encryption and decryption.

### 4.2. Key Sensitivity Analysis

A desirable property of a secure encryption algorithm is high key sensitivity, such that even a negligible perturbation in the key produces completely different encryption or decryption outcomes. In the proposed algorithm, the permutation and diffusion operations are jointly governed by the initial conditions and control parameters of the chaotic system. Due to the inherent sensitivity of chaotic dynamics to initial conditions, a tiny perturbation in the key can cause substantial changes in the generated chaotic sequences, resulting in completely different ciphertexts and unsuccessful decryption.

**Figure 7:**
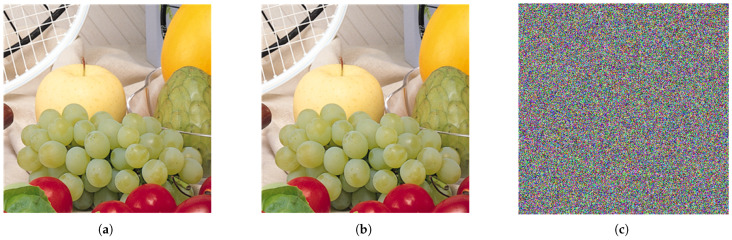

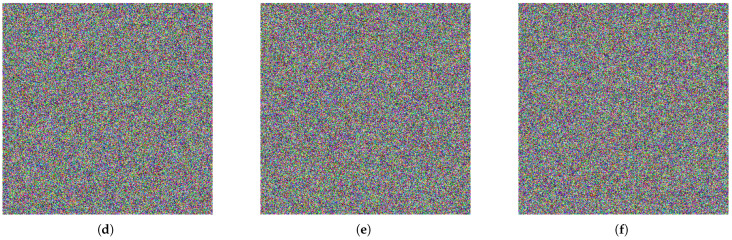
Key sensitivity test results of the proposed algorithm for the Fruits image. (**a**) Plaintext image; (**b**) decrypted result using the correct key; (**c**) decrypted result with $x_{0}+10^{-14}$; (**d**) decrypted result with $y_{0}+10^{-14}$; (**e**) decrypted result with $z_{0}-10^{-14}$; and (**f**) decrypted result with $m-10^{-14}$.

To evaluate the key sensitivity of the proposed algorithm, the 512×512 Fruits image was selected for testing. Decryption was first carried out with the correct key and then repeated using four slightly modified keys generated by perturbing $x_{0}$, $y_{0}$, $z_{0}$, and *m* by $10^{-14}$, respectively. The corresponding results are shown in Figure 7. It can be seen that the plaintext image can be correctly recovered only when the exact key is used, whereas even a negligible change in any of these parameters leads to decryption failure. These results confirm that the proposed algorithm possesses strong key sensitivity.

### 4.3. Histogram Analysis

**Figure 8:**
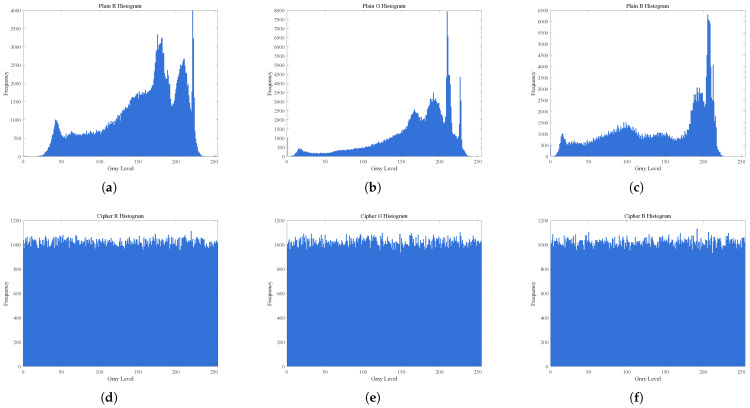
Histogram comparison of the RGB channels for the plaintext image and the corresponding cipher image: (**a**) plaintext-image R channel, (**b**) plaintext-image G channel, (**c**) plaintext-image B channel, (**d**) cipher image R channel, (**e**) cipher image G channel, and (**f**) cipher image B channel.

Histogram analysis is commonly used to evaluate whether an encryption algorithm can effectively conceal the statistical characteristics of a plaintext image. Figure 8 shows the histograms of the House image before and after encryption for the three color channels. The histograms of the plaintext image are evidently nonuniform and contain several pronounced peaks, particularly in the high gray-level regions, indicating considerable statistical redundancy in the plaintext image. After encryption, however, the histograms of the R, G, and B channels become substantially flatter and more evenly distributed, and the dominant peaks disappear. This behavior indicates that the proposed algorithm can effectively conceal the statistical distribution of pixel values and significantly enhance the randomness of the encrypted image, which is beneficial for resisting statistical attacks.

### 4.4. Correlation Analysis

**Figure 9:**
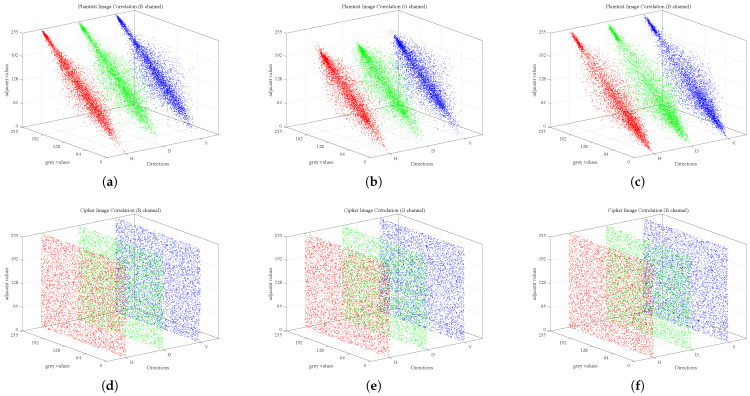
Correlation plots of the plaintext and cipher Baboon image. (**a**–**c**) correlation plots of the plaintext RGB components; (**d**–**f**) correlation plots of the cipher image RGB components.

In plaintext images, adjacent pixels generally exhibit strong correlation in the horizontal, vertical, and diagonal directions. An effective image encryption algorithm should therefore disrupt this intrinsic dependency and render neighboring pixels statistically independent. To evaluate the decorrelation capability of the proposed method, the correlation coefficients of adjacent pixels in the Baboon image were calculated for the R, G, and B channels along the three aforementioned directions. The corresponding scatter plots and numerical results are provided in Figure 9 and Table 2, respectively. As can be observed from Figure 9a–c, the adjacent pixel pairs in the plaintext image are tightly clustered around the diagonal, reflecting the strong correlation naturally present in unencrypted images. By contrast, the scatter distributions of the encrypted image shown in Figure 9d–f are highly dispersed and exhibit no clear structural pattern, suggesting that the dependence between neighboring pixels has been effectively eliminated. This conclusion is further supported by the data in Table 2, where the correlation coefficients of the cipher image fall within the interval from −0.0014 to 0.0050, all of which are very close to zero. These results demonstrate that the proposed algorithm has a strong ability to eliminate adjacent-pixel correlation and thus provides effective protection against statistical analysis.

### 4.5. Information Entropy Analysis

Information entropy is a standard metric for measuring the randomness of encrypted images. For an 8-bit image, the theoretical entropy of each channel is 8. A value closer to 8 indicates a more uniform pixel distribution and weaker statistical characteristics in the cipher image. It is given by

(65) $$H\left(m\right)=-\sum_{i=0}^{255}P\left(m_{i}\right)\log_{2}P\left(m_{i}\right)$$

where $P(m_{i})$ denotes the probability of gray value $m_{i}$, and $\log_{2}\left(\cdot \right)$ is used for 8-bit entropy calculation. To evaluate the statistical security of the proposed algorithm, the Baboon image was selected for testing. The results are listed in Table 3.

As shown in Table 3, the proposed algorithm yields entropy values very close to the theoretical limit in all three channels. Its performance is comparable to, and in some cases better than, those of the reference algorithms. This indicates that the proposed algorithm can effectively eliminate the statistical structure of the plaintext image and generate cipher images with high randomness, thereby improving resistance to statistical attacks.

### 4.6. Differential Attack Analysis

Differential attack analysis is an important criterion for evaluating the sensitivity of an image encryption algorithm to slight changes in the plaintext. A robust encryption algorithm should ensure that even a one-pixel modification in the plaintext causes significant and widespread changes in the corresponding ciphertext. In this paper, the Number of Pixels Change Rate (NPCR) and the Unified Average Changing Intensity (UACI) are employed for quantitative evaluation. Let $C_{1}$ and $C_{2}$ denote two M×N cipher images obtained by encrypting two plaintext images that differ only slightly. The corresponding metrics are defined as follows.

(66) $$\left\{\begin{array}{cc} NPCR & =\frac{1}{M\times N}\sum_{i=1}^{M}\sum_{j=1}^{N}D\left(i,j\right)\times 100\%\\ UACI & =\frac{1}{M\times N}\sum_{i=1}^{M}\sum_{j=1}^{N}\frac{|C_{1}\left(i,j\right)-C_{2}\left(i,j\right)|}{255}\times 100\% \end{array}\right.$$

For 8-bit images, the theoretical ideal values of NPCR and UACI are approximately 99.6094% and 33.4635%, respectively. The proposed algorithm strengthens nonlinear confusion through dynamic S-box substitution and accelerates disturbance propagation among RGB channels by means of a forward-backward cross-channel diffusion mechanism. As shown in Table 4, for the Baboon image, the NPCR values of the R, G, and B channels are 99.6063%, 99.6158%, and 99.6052%, respectively, while the corresponding UACI values are 33.4947%, 33.5159%, and 33.4486%. These results are very close to the theoretical values and are comparable to, and in some cases better than, those of the reference algorithms, indicating that the proposed algorithm exhibits high sensitivity to slight plaintext changes and strong resistance to differential attacks.

### 4.7. Robustness Analysis

In practical image transmission, cipher images may suffer from data loss, channel noise, or intentional tampering. Although an unauthorized attacker cannot recover the plaintext without the correct key, ciphertext degradation may still affect the quality of the decrypted results. Therefore, it is necessary to evaluate the robustness of the proposed algorithm under typical ciphertext degradation scenarios.

In this section, cropping attack, salt-and-pepper noise attack, and Gaussian noise attack are considered. The experimental procedure is as follows. First, the original image is encrypted to obtain the cipher image. Then, different attacks are applied to the cipher image. Finally, the attacked cipher image is decrypted using the correct key, and the recovered image is compared with the original image.

In this study, peak signal-to-noise ratio (PSNR) and bit correct rate (BCR) are adopted as the main quantitative metrics. Specifically, PSNR is mainly reported for cropping attacks to evaluate the visual reconstruction quality of the recovered images, whereas BCR is mainly reported for noise attacks to quantify the bit-level consistency between the recovered images and the original images. The mean square error, MSE, is defined as

(67) $$MSE=\frac{1}{3MN}\sum_{\chi \in \{R,G,B\}}\sum_{i=1}^{M}\sum_{j=1}^{N}{\left[I_{\chi }\left(i,j\right)-D_{\chi }\left(i,j\right)\right]}^{2}$$

where *I* and *D* denote the original image and the decrypted image from the attacked ciphertext, respectively. M×N is the image size, and χ represents the color channel.

The PSNR is defined as

(68) $$PSNR=10\log_{10}\left(\frac{255^{2}}{MSE}\right)$$

A higher PSNR value indicates better reconstruction quality.

The BER is defined as

(69) $$BER=\frac{N_{\mathrm{err}}}{N_{\mathrm{total}}}$$

where $N_{\mathrm{err}}$ and $N_{\mathrm{total}}$ represent the number of erroneous bits and the total number of bits, respectively. Accordingly, BCR is given by

(70) BCR=1−BER

A BCR value closer to 1 indicates higher bit-level consistency between the recovered image and the original image.

#### 4.7.1. Cropping Attack

**Figure 10:**
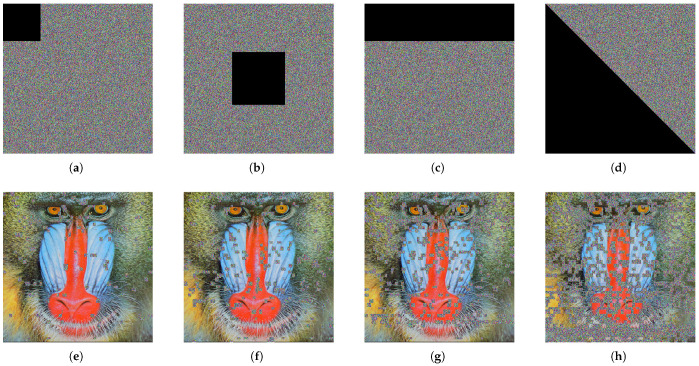
Decryption results under different cropping attacks: (**a**) upper-left cropping, 6.25%; (**b**) central cropping, 12.5%; (**c**) top-strip cropping, 25%; (**d**) diagonal triangular cropping, 50%; (**e**–**h**) the corresponding decrypted images.

To evaluate the robustness against local ciphertext loss, four cropping patterns were applied to the cipher images, followed by decryption with the correct key. The visual and quantitative results are shown in Figure 10 and Table 5, respectively.

As shown in Figure 10, cropping attacks introduce different levels of distortion into the decrypted images. As the cropped area increases, the degradation becomes more evident. Nevertheless, the main visual content and structural contours of the recovered images remain recognizable even under severe cropping conditions.

The PSNR results in Table 5 show that the proposed algorithm exhibits a more gradual degradation trend than the compared algorithms as the cropping ratio increases. Although Algorithm [42] obtains a slightly higher PSNR under the 6.25% cropping attack, its performance drops sharply when the cropping ratio increases. In contrast, the proposed algorithm maintains competitive and relatively stable reconstruction quality under increasing cropping ratios. This demonstrates that the proposed algorithm has comparatively better robustness against ciphertext data loss.

#### 4.7.2. Salt-and-Pepper Noise Attack

**Figure 11:**
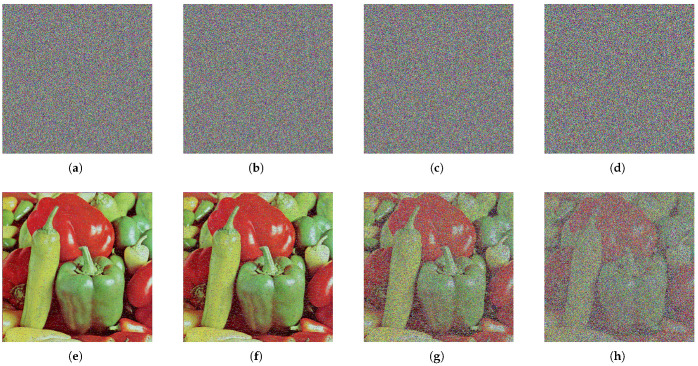
Decryption results of the proposed algorithm under salt-and-pepper noise attacks. (**a**–**d**) Cipher images contaminated with salt-and-pepper noise at densities of 0.01, 0.02, 0.1, and 0.2, respectively; (**e**–**h**) the corresponding decrypted images.

To evaluate the robustness against impulse noise, salt-and-pepper noise with densities of 0.01, 0.02, 0.10, and 0.20 was added to the cipher images. The contaminated cipher images were then decrypted using the correct key. The visual results are shown in Figure 11.

At low noise densities, the recovered images preserve most visual details and exhibit only slight degradation. As the noise density increases, random black-and-white impulses cause more severe corruption in the decrypted images. However, the dominant image regions and basic structural information remain distinguishable. This suggests that the proposed algorithm can tolerate a certain degree of impulse noise in the ciphertext and still retain useful visual information.

#### 4.7.3. Gaussian Noise Attack

**Figure 12:**
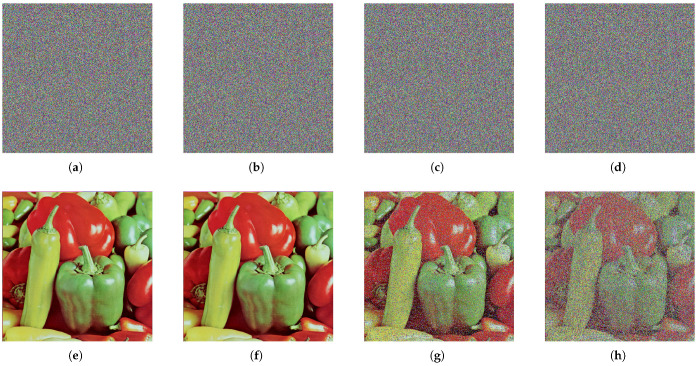
Decryption results of the proposed algorithm under Gaussian noise attacks. (**a**–**d**) Cipher images contaminated with Gaussian noise with variances of $1\times 10^{-6}$, $2\times 10^{-6}$, $1\times 10^{-5}$, and $2\times 10^{-5}$, respectively; (**e**–**h**) the corresponding decrypted images.

Gaussian noise with variances of $1\times 10^{-6}$, $2\times 10^{-6}$, $1\times 10^{-5}$, and $2\times 10^{-5}$ was added to the cipher images to model channel noise contamination. Figure 12 illustrates the decrypted images obtained from the noisy ciphertexts.

Different from salt-and-pepper noise, Gaussian noise introduces continuous perturbations into the cipher images. As the noise variance increases, the recovered images become increasingly blurred and distorted. Even so, the principal scene information remains observable at the higher tested noise variances. This indicates that the proposed algorithm can still retain essential visual content under Gaussian channel noise interference.

The quantitative BCR results for the Peppers image under the two noise attacks are summarized in Table 6. For RGB images, BCR is calculated separately for the red, green, and blue channels. Under salt-and-pepper noise, the BCR values decrease steadily as the noise density increases from 0.01 to 0.20. Specifically, the BCR values decrease from approximately 0.9958 to about 0.654. Under Gaussian noise, the BCR values remain nearly unchanged at low variances but drop noticeably when the variance increases to $1\times 10^{-5}$, and $2\times 10^{-5}$. These results confirm that stronger noise interference reduces bit-level reconstruction accuracy. Nevertheless, together with the visual results in Figure 11 and Figure 12, the BCR values indicate that the proposed algorithm can still preserve useful visual information under both impulse noise and Gaussian channel noise.

### 4.8. Efficiency Analysis

The efficiency of an image encryption algorithm is important for practical applications, especially in real-time transmission and resource-constrained scenarios. In this section, the efficiency of the proposed algorithm is evaluated in terms of execution time and computational cost.

#### 4.8.1. Execution Time Analysis

To evaluate the execution efficiency of the proposed algorithm, encryption and decryption experiments were conducted under the experimental environment described at the beginning of this chapter. The Lena color image with two different sizes, namely 256×256×3 and 512×512×3, was used for testing. Each experiment was independently repeated 20 times, and the average running time was recorded. The results are listed in Table 7.

As shown in Table 7, for both Lena image sizes, the proposed algorithm requires less time for encryption and decryption than the compared methods.

#### 4.8.2. Computational Complexity Analysis

Computational complexity is evaluated by considering both the estimated number of pixel-level operations and the asymptotic time complexity. For an RGB image of size M×N×3, the proposed algorithm mainly includes chaotic sequence generation, dynamic rectangular partitioning, inter-block permutation, intra-block permutation, dynamic S-box substitution, and bidirectional diffusion.

The estimated costs of these operations are MN, MN, MN, MN, 3MN, and 6MN, respectively. Therefore, the total estimated computational cost is

(71) MN+MN+MN+MN+3MN+6MN=13MN

Since constant coefficients are ignored in asymptotic complexity analysis, the asymptotic time complexity of the proposed algorithm is O(MN).

Table 8 compares the computational costs of different algorithms. Although the compared algorithms have the same asymptotic order of O(MN), the proposed algorithm has a lower estimated computational cost in terms of the number of pixel-level operations.

In terms of memory consumption, the proposed algorithm mainly requires storage for the input/output RGB images, image-scale intermediate arrays generated during permutation and diffusion, and chaotic control sequences used in different encryption stages. Since these data structures are all proportional to the image size, the memory requirement increases linearly with the number of pixels. Therefore, for an RGB image of size M×N×3, the memory complexity of the proposed algorithm is O(MN).

## 5. Conclusions

In this paper, a color image encryption method based on 3D-SBFCM, dynamic rectangular partitioning, and dynamic S-box substitution is proposed. The combination of chaotic dynamics, adaptive partitioning, and nonlinear substitution enhances the randomness and security of encrypted images. Experimental results verify the effectiveness and robustness of the proposed algorithm. In future research, the dynamic rectangular partitioning process will be further refined to enhance its adaptability to images with different textures and structures, while the dynamic S-box construction mechanism will be optimized to further strengthen the adaptability and security of the algorithm.

## Figures and Tables

**Table 1:** NIST test values for 3D-SBFCM (P: Pass; F: Fail).

Test Item	*p* Value (x)	*p* Value (y)	*p* Value (z)	Results
Frequency	0.7733	0.1929	0.3513	Pass
Block Frequency	0.5785	0.1377	0.7387	Pass
Runs	0.5208	0.8731	0.5358	Pass
Longest Run of Ones	0.2942	0.8238	0.9236	Pass
Binary Matrix Rank	0.2221	0.2163	0.6639	Pass
Discrete Fourier Transform	0.8803	0.3127	0.9141	Pass
Non-Overlapping Template	0.5068	0.5032	0.4993	Pass
Overlapping Matching	0.9661	0.9969	0.9937	Pass
Universal Statistical	0.7717	0.8924	0.2446	Pass
Linear Complexity	0.2582	0.9793	0.1456	Pass
Serial	0.1655	0.2134	0.0982	Pass
Approximate Entropy	0.9172	0.0465	0.8234	Pass
Cumulative Sums	0.4185	0.1014	0.3286	Pass
Random Excursion	0.5638	0.2365	0.6063	Pass
Random Excursion Variant	0.0397	0.3244	0.3753	Pass

**Table 2:** Comparison of correlation coefficients for the encrypted Baboon image.

Channels	Directions	Proposed Algorithm	Algorithm [36]	Algorithm [37]	Algorithm [38]
R	H	−0.0013	0.0085	0.0018	0.0009
	V	0.0050	−0.0025	−0.0050	−0.0028
	D	0.0007	−0.0151	−0.0096	0.0015
G	H	0.0016	0.0009	0.0043	0.0025
	V	0.0025	−0.0058	−0.0055	−0.0020
	D	−0.0014	−0.0007	0.0061	−0.0020
B	H	0.0020	0.0045	−0.0015	−0.0004
	V	0.0023	−0.0084	−0.0030	0.0010
	D	0.0009	−0.0048	−0.0029	−0.0028

**Table 3:** Information entropy of encrypted Baboon image.

Images	Proposed Algorithm	Algorithm [36]	Algorithm [37]	Algorithm [39]	Algorithm [40]
R channel	7.9993	7.9972	7.9993	7.9992	7.9915
G channel	7.9994	7.9975	7.9993	7.9993	7.9913
B channel	7.9993	7.9971	7.9993	7.9993	7.9914

**Table 4:** NPCR and UACI results of the Baboon image for different encryption algorithms.

Images	NPCR			UACI		
	R	G	B	R	G	B
Proposed algorithm	99.6063	99.6158	99.6052	33.4947	33.5159	33.4486
Algorithm [39]	99.6197	99.6181	99.6250	33.4954	33.4996	33.5360
Algorithm [40]	99.6132	99.5995	99.6109	33.4543	33.4034	33.4275
Algorithm [41]	99.6114	99.6246	99.6195	33.4247	33.4585	33.4619

**Table 5:** PSNR comparison under cropping attacks.

Algorithm	Cropping Attack (PSNR)			
	6.25%	12.5%	25%	50%
Proposed algorithm	20.7754	17.9212	15.6189	13.5742
Algorithm [42]	20.8054	15.6806	14.7826	7.7850
Algorithm [43]	20.6616	17.8724	15.2783	13.3415
Algorithm [44]	19.9545	17.3595	14.3256	12.2368

**Table 6:** Channel-wise BCR robustness analysis of the Peppers image under noise attacks.

Noise Type	Noise Parameter	Red Channel	Green Channel	Blue Channel
SPN	0.01	0.9958	0.9956	0.9958
	0.02	0.9653	0.9647	0.9652
	0.10	0.7989	0.7988	0.7988
	0.20	0.6540	0.6546	0.6542
GN	$1\times 10^{-6}$	0.9999	0.9999	0.9999
	$2\times 10^{-6}$	0.9999	0.9999	0.9999
	$1\times 10^{-5}$	0.8526	0.8521	0.8526
	$2\times 10^{-5}$	0.6864	0.6809	0.6806

**Table 7:** Execution time comparison of different encryption algorithms (Unit: s).

Image Size	Process	Proposed Algorithm	Algorithm [31]	Algorithm [45]
256×256×3	Encryption	0.9654	0.9925	2.0151
	Decryption	0.9347	0.9682	2.6891
512×512×3	Encryption	2.9875	4.0036	6.4257
	Decryption	2.6512	3.4552	9.4804

**Table 8:** Computational complexity comparison of different encryption algorithms.

Algorithms	Estimated Computational Cost	Asymptotic Complexity
Proposed algorithm	13MN	O(MN)
Algorithm [42]	16MN	O(MN)
Algorithm [46]	20MN	O(MN)
Algorithm [47]	61MN+3(M+N)	O(MN)

## Data Availability

The standard test images used in this study are publicly available from commonly used image processing benchmark databases. The experimental results generated during this study are available from the corresponding author upon reasonable request.
